# Symptom Clusters and Frailty Among Older Adults With and Without Cancer: Insights From Two Cross‐Sectional Studies

**DOI:** 10.1111/nhs.70406

**Published:** 2026-08-02

**Authors:** Tianxue Hou, Denise Shuk Ting Cheung, Mu‐Hsing Ho, Tongyao Wang, Chia‐Chin Lin

**Affiliations:** ^1^ School of Nursing, Li Ka Shing Faculty of Medicine, the University of Hong Kong Pok Fu Lam Hong Kong; ^2^ Alice Ho Miu Ling Nethersole Charity Foundation Tai Po New Territories Hong Kong; ^3^ Research Unit for Enhancing Well‐Being in Vulnerable and Chronic Illness Populations, Faculty of Nursing Chulalongkorn University Bangkok Thailand

**Keywords:** depressive symptoms, frailty, older adults, pain, sleep disturbances, symptom clusters

## Abstract

This study aims to examine the relationship between symptom clusters (depressive symptoms, sleep disturbances, and pain) and frailty in older adults, with a specific focus on mediation and moderation effects. Data from two cross‐sectional studies in Changsha and Hong Kong were analyzed, including nursing home residents without cancer and older adults with cancer. Study‐stratified analyses were conducted in SPSS 26.0 and PROCESS, with adjustment for available covariates. Mediation tested whether sleep disturbances were indirectly associated with depressive symptoms and frailty, and moderated mediation examined whether pain severity or interference conditioned the association between depressive symptoms and sleep disturbances. In both groups, frailty, depressive symptoms, sleep disturbances, and pain were significantly associated. Sleep disturbances showed a significant indirect association between depressive symptoms and frailty in both groups. Pain interference, but not pain severity, moderated the association between depressive symptoms and sleep disturbances only among older adults without cancer. The findings highlight the importance of assessing and managing depressive symptoms, sleep disturbances, and pain‐related interference when addressing frailty in older adults, particularly among those without cancer.

## Key Points

- Frailty is associated with depressive symptoms, sleep disturbance, and pain.
- Sleep disturbances show a significant indirect association between depressive symptoms and frailty in both older adults with and without cancer, suggesting a shared pattern across populations.
- Pain interference moderates the association between depressive symptoms and sleep disturbances, but only among older adults without cancer.

## 1 Introduction

Frailty is a clinical syndrome characterized by diminished physical strength, reduced endurance, and functional decline (Morley et al. 2013), which significantly affects older individuals, particularly those in long‐term care facilities (Collard et al. 2012; Kojima 2015). Older cancer survivors are also vulnerable to frailty due to the cumulative effects of cancer treatment and disease‐related factors, which can exacerbate physical decline compared to their non‐cancer counterparts (Zhang et al. 2022). Multiple factors influence this condition and increase vulnerability to falls, disability, hospitalization, and mortality (Boyle et al. 2010; Fried et al. 2001). Identifying modifiable risks, including co‐occurring symptoms, is crucial for developing interventions to slow frailty progression (Coleman et al. 2023; Yuan and Zhang 2023).

### 1.1 Symptom Clusters

Older individuals often experience overlapping symptoms regardless of cancer history. A symptom cluster refers to a group of interconnected symptoms that may share underlying mechanisms or collectively affect health outcomes (Miaskowski et al. 2004). This concept aligns with the Theory of Unpleasant Symptoms, which suggests that symptoms interact through complex pathways to influence health conditions, such as mediation or moderation (Barsevick et al. 2006; Lenz et al. 1997, 1995). Some studies demonstrate these dynamics; for example, pain may exacerbate depressive symptoms, which in turn accelerate frailty (Tian et al. 2018), while sleep disturbances can exacerbate both psychological distress and physical decline (X. Liu, Wang, et al. 2021). These findings highlight the importance of examining symptom interactions rather than individual symptoms.

### 1.2 The Triad of Depressive Symptoms, Sleep Disturbances, and Pain

Depressive symptoms, sleep disturbances, and pain frequently co‐occur in older adults with and without cancer (Bao et al. 2017; X. Liu, Wang, et al. 2021). A study of 120 older patients with cancer revealed that about 20% reported two concurrent symptoms (e.g., depression and pain), while 31.2% experienced all four symptoms of depression, pain, sleep issues, and fatigue (Cheng and Lee 2011). Similarly, 76.7% of nursing home residents without cancer reported at least two co‐occurring symptoms (Hou 2024), and a systematic review identified a 39.6% prevalence of depressive symptoms in older adults with sleep disturbance (Bao et al. 2017). While many studies focus on pain severity as a primary component of symptom clusters, the impact of pain on daily functioning is equally crucial (Beck et al. 2005). Pain interference refers to the effects of pain on daily functioning, which plays a significant role in symptom clusters. Research indicates that pain interference, rather than pain intensity alone, influences psychosocial outcomes and functional decline (Wicksell et al. 2016), emphasizing the need to address both symptom severity and its daily impact. Given the distinct physiological and psychological impacts of cancer and its treatments, it is important to investigate how symptom clusters affect frailty differently in older adults with and without cancer (Antoni et al. 2023).

### 1.3 Interconnected Pathways to Frailty

Emerging evidence highlights bidirectional relationships among these symptoms. Sleep disturbances impair hormonal regulation (e.g., testosterone and growth hormone), accelerating muscle loss and frailty (Wai and Yu 2020), while simultaneously exacerbating depressive symptoms (Chen et al. 2023). Conversely, depressive symptoms may impair sleep quality, creating a cyclical pattern (Hui et al. 2021). Pain further complicates this triad by correlating with both psychological distress and sleep disruption (Blytt et al. 2021; M. Liu, Hou, et al. 2021), and shared biological pathways (e.g., neurotransmitter dysregulation) underpin the connection between pain and depressive symptoms (Blier and Abbott 2001; Chopra and Arora 2014). This interplay suggests that pain may moderate the effects of depressive symptoms on sleep disturbances, ultimately influencing frailty risk (Keilani et al. 2018; Orzeszek et al. 2024).

### 1.4 Research Gaps

Despite increasing recognition of the associations between depressive symptoms, sleep disturbances, pain, and frailty, some gaps remain. Prior studies often focus on isolated symptoms or single populations, limiting understanding of how these symptoms interact as pathways to frailty. It is also unclear whether symptom‐frailty associations are consistent across older adults with and without cancer. Furthermore, the distinct contributions of pain severity and pain interference to these associations have rarely been examined. Addressing these gaps is important for identifying both common and population‐specific symptom patterns that may provide insights for tailored frailty management.

### 1.5 Research Hypotheses

**FIGURE 1:**
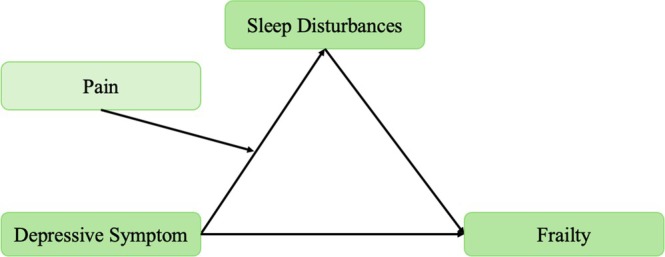
Theoretical model.

We proposed the following hypotheses (Figure 1):

Hypothesis 1 *Sleep disturbances mediate the relationship between depressive symptoms and frailty among older adults with and without cancer*.

Hypothesis 2 *Pain interference, rather than pain severity, moderates the association between depressive symptoms and sleep disturbances in older adults with and without cancer*.

## 2 Methods

### 2.1 Aims

This study aims to address these limitations by using data from two cross‐sectional studies to compare how sleep disturbances and pain (severity and interference) interact with depressive symptoms to influence frailty in older adults with and without cancer. By identifying these mechanisms, our findings aim to provide insights for multifactorial interventions to delay or prevent frailty in vulnerable aging populations.

### 2.2 Study Design

We utilized two cross‐sectional datasets from Changsha and Hong Kong, China. One dataset comprised nursing home residents without cancer, a population characterized by high rates of frailty and co‐occurring symptoms due to long‐term care needs. The other dataset included older patients with cancer recruited from outpatient oncology clinics, where ongoing assessment and symptom management are routinely provided. This design reflects real‐world care pathways, enabling comparison of symptom‐frailty associations across distinct populations.

#### 2.2.1 Study 1—Changsha

##### 2.2.1.1 Participants and Procedures

Participants were recruited using a convenience sampling approach from 10 nursing homes in Changsha between 2021 and 2022. Data were collected through interviewer‐administered questionnaires in a private setting. Eligible participants were aged 65 years or older without cancer, had resided in a nursing home for over 6 months (Lim et al. 2022), and were classified as having normal cognitive function based on past medical records. Exclusion criteria included severe sensory impairments, unconsciousness, or mental illnesses that could affect cognitive function. Facility staff screened eligibility using medical records. Trained research assistants then approached eligible residents, provided study information, and obtained written informed consent.

##### 2.2.1.2 Questionnaire

The questionnaire comprised three parts: (1) Demographic characteristics (age, gender, BMI, education level, and marital status); (2) the FRAIL‐NH scale, which assesses seven domains of frailty (fatigue, transferring, mobility, incontinence, weight loss, nutrition, and dressing) with a total score ranging from 0 to 14 (Kaehr et al. 2015). The Cronbach's *α* was found to be 0.69; and (3) symptom clusters include (1) Patient Health Questionnaire‐9 (PHQ‐9), which assesses depressive symptoms severity over the past 2 weeks with scores ranging from 0 to 27, where 0–4 indicates none or minimal depression and over 5 indicates depression (Belanger and Winsberg 2022; Kroenke et al. 2001). The Cronbach's *α* value was 0.76. (2) The Pittsburgh Sleep Quality Index (PSQI) assesses sleep quality using 19 self‐assessment items and 5 additional items, covering components like subjective sleep quality, sleep latency, sleep duration, sleep efficiency, sleep disturbance, use of sleep medication, and daytime dysfunction, with scores ranging from 0 to 21 (Buysse et al. 1989; Luyster et al. 2015). The Cronbach's *α* value was 0.80; and (3) The Brief Pain Inventory‐short form (BPI‐sf) evaluates pain severity and its interference with daily functions, including categories like “worst,” “least,” “average” over the past 24 h, and “pain now,” as well as how pain interferes with “enjoyment of life,” “general activity,” “walking ability,” “mood,” “sleep,” “normal work,” and “relations with others,” with overall pain intensity and impact determined by averaging the respective scores (Cleeland and Ryan 1994; Poquet and Lin 2016). The Cronbach's *α* value was 0.94.

#### 2.2.2 Study 2‐Hong Kong

##### 2.2.2.1 Participants and Procedures

Older patients with cancer were recruited from one oncology outpatient clinic in Hong Kong between 2019 and 2021 using a convenience sampling strategy. Clinic staff identified potentially eligible patients according to age and diagnosis, and research assistants then confirmed eligibility, explained the study, and obtained written informed consent prior to questionnaire administration. Eligible individuals were aged 65 years or older and diagnosed with cancer. Exclusion criteria included inability to communicate in Cantonese or Mandarin or inability to provide written informed consent.

##### 2.2.2.2 Questionnaire

The questionnaire comprised four parts: (1) demographic characteristics (age, gender, education level, marital status, and cancer type); (2) the FRAIL scale, which evaluates five components (fatigue, resistance, ambulation, illnesses, and weight loss) with scores ranging from 0 to 5 (Morley et al. 2012). The Cronbach's *α* value was 0.70; and (3) symptom clusters: (1) The Geriatric Depression Scale‐15 item (GDS‐15) assesses depressive symptoms with scores ranging from 0 to 15 (0–4 normal, over 5 indicating depression) (Almeida and Almeida 1999). The Cronbach's *α* value was 0.78. (2) The EORTC Core Quality of Life questionnaire (EORTC QLQ‐C30) assesses health‐related quality of life with 30 items, including 5 functional subscales, 9 symptom subscales, and 1 global health‐status scale. It also includes six single items assessing dyspnea, appetite loss, sleep disturbance, constipation, diarrhea, and financial impact (Fayers et al. 2001). In this study, this scale was specifically utilized to measure sleep disturbances and pain (pain severity and pain interference).

### 2.3 Statistical Analysis

Given the distinct sites and care contexts, analyses were performed separately for each study to examine within‐sample associations and hypothesized pathways, rather than direct comparison of symptom scores across sites. Descriptive statistics were used to summarize demographic and outcome variables. Categorical variables were presented as frequency (*n*) and percentage (%), while continuous variables were expressed as mean ± standard deviation (*M* ± SD). Chi‐square tests were used to compare demographic and outcome variables between the two groups. Spearman correlation analysis was performed to examine associations among depressive symptoms, sleep disturbances, pain, and frailty within each population.

Each study employed validated instruments suited to its clinical context. To enhance comparability across instruments, all continuous variables were standardized using *z*‐score transformation within each study before mediation and moderation analyses. Analyses focused on replication of within‐sample patterns rather than direct comparison of absolute scores across sites.

Data analysis was conducted using SPSS version 26.0 and PROCESS v4.2. Mediation and moderated mediation effects were examined with Hayes' PROCESS macro (version 4.2), applying bias‐corrected bootstrapping (2000 resamples) to estimate 95% confidence intervals (Hayes 2013). In line with study hypotheses, Model 4 was used to examine whether sleep disturbances showed an indirect association between depressive symptoms and frailty. Model 7 was used to test whether pain moderated the association between depressive symptoms and sleep disturbances. Pain severity and pain interference were analyzed separately to distinguish symptom intensity from functional impact. Sleep disturbances were assessed with the PSQI in Study 1 and with the sleep disturbance item of the EORTC QLQ‐C30 in Study 2. In Study 2, pain severity and pain interference were assessed using the corresponding pain items of the EORTC QLQ‐C30. Sociodemographic and health‐related covariates available in both studies (e.g., age, sex, education level, marital status, and BMI) were included as covariates in the PROCESS analyses. No missing values were identified among the variables included in the mediation and moderated mediation analyses.

### 2.4 Common Method Deviation Test

To account for the potential common method bias effect resulting from self‐reported data, Harman's one‐way analysis was employed in these two studies (Tehseen et al. 2017). The items underwent unrotated principal component factor analysis, and the first factor accounted for 21.22% and 22.97% of the variance for the two studies, respectively, which is below the threshold of 40%. This suggests that the data collected in these studies did not exhibit a significant common method bias.

## 3 Results

### 3.1 Descriptive Statistics

The analytic sample included 172 nursing home residents without cancer in Study 1 and 227 older patients with cancer in Study 2. On average, nursing home residents in Study 1 were older (81.76 vs. 71.04 years) and had a lower BMI (22.31 vs. 23.45) compared with participants in Study 2. Study 1 participants were also less likely to be married (17.4% vs. 63.0%) and had a lower educational level, with fewer having completed high school or above (37.2% vs. 60.7%). Smoking was more common among nursing home residents than among older patients with cancer (9.3% vs. 1.8%, *p* = 0.001), whereas alcohol use showed no statistically significant difference between the two groups (4.7% vs. 7.0%, *p* = 0.21). In Study 2, the most common cancer type was breast cancer (49.3%), followed by colorectum (14.5%), prostate (11.9%), and lung cancer (8.8%). Nearly all older patients with cancer had received treatment (99.6%) (Table 1).

**TABLE 1:** Results of descriptive statistics (*N* = 399).

Variables	Nursing home residents (*n* = 172), *M* ± SD, *n* (%)	Older cancer patients (*n* = 227), *M* ± SD, *n* (%)	*p*
Age	81.76 ± 9.26	71.04 ± 4.46	< 0.001
Gender			
Female	112 (65.1)	165 (72.7)	0.10
Male	60 (34.9)	62 (27.3)	
BMI	22.31 ± 3.54	23.45 ± 3.85	0.02
Marital status			
Married	30 (17.4)	143 (63.0)	< 0.001
Unmarried	5 (2.9)	33 (14.5)	
Separated/divorce/widowed	2137 (79.7)	51 (22.5)	
Education level			
Less than high school	108 (62.8)	89 (39.2)	< 0.001
High school graduates	36 (20.9)	85 (37.4)	
College or higher	28 (16.3)	53 (23.3)	
Smoking			
No	156 (90.7)	223 (98.2)	0.001
Yes	16 (9.3)	4 (1.8)	
Drinking			
No	164 (95.3)	211 (93)	0.21
Yes	8 (4.7)	16 (7)	
Depressive symptoms			
No	39 (22.7)	187 (82.4)	< 0.001
Yes	133 (77.3)	40 (17.6)	
Sleep disturbances score	1.72 ± 0.74	1.79 ± 0.91	0.39
Pain severity score	1.39 ± 1.42	1.59 ± 0.68	< 0.001
Pain interference score	1.13 ± 1.48	1.38 ± 0.66	0.02
Frailty score	3.2 ± 2.36	1.35 ± 0.77	< 0.001
Cancer type			
Colorectum		33 (14.5)	
Lung		20 (8.8)	
Breast		112 (49.3)	
Prostate		27 (11.9)	
Others		35 (15.4)	
Cancer stage			
Stage 0		44 (19.4)	
Stage 1		69 (30.4)	
Stage 2		58 (25.6)	
Stage 3		40 (17.6)	
Stage 4		15 (7)	
Received treatment			
No		1 (0.4)	
Yes		226 (99.6)	

### 3.2 Correlations Between Depressive Symptoms, Sleep Disturbances, Pain, and Frailty

Table 2 presents study‐stratified correlations. The measures reflect the instruments used in each study (PHQ‐9, PSQI, BPI, and FRAIL‐NH in Study 1; GDS‐15, EORTC QLQ‐C30, and FRAIL in Study 2) and are interpreted within each study. These correlations showed statistically significant associations within each study, supporting subsequent analyses of mediation and moderated mediation.

**TABLE 2:** Descriptive statistics and correlations between variables in nursing home residents and older cancer patients (*N* = 399).

Variables	1	2	3	4	5
*Nursing home residents (n = 172)*					
1 Frailty	1.00				
2 Depressive Symptoms	0.35**	1.00			
3 Sleep Disturbance	0.36**	0.33**	1.00		
4 Pain Severity	0.20**	0.26**	0.54**	1.00	
5 Pain Interference	0.31**	0.25**	0.60**	0.87**	1.00
*Older cancer patients (n = 227)*					
1 Frailty	1.00				
2 Depressive Symptoms	0.19**	1.00			
3 Sleep Disturbance	0.17**	0.21**	1.00		
4 Pain Severity	0.10	0.33**	0.21**	1.00	
5 Pain Interference	0.12	0.37**	0.17*	0.63**	1.00

* *p* < 0.05.

** *p* < 0.01.

In Study 1, FRAIL‐NH scores were positively correlated with PHQ‐9 scores (*r* = 0.35, *p* < 0.01), PSQI scores (*r* = 0.36, *p* < 0.01), BPI pain severity (*r* = 0.20, *p* < 0.01), and BPI pain interference (*r* = 0.31, *p* < 0.01). PHQ‐9 scores were correlated with PSQI scores (*r* = 0.33, *p* < 0.01), BPI pain severity (*r* = 0.26, *p* < 0.01), and BPI pain interference (*r* = 0.25, *p* < 0.01). Additionally, PSQI scores were strongly associated with BPI pain severity (*r* = 0.54, *p* < 0.01) and BPI pain interference (*r* = 0.60, *p* < 0.01).

In Study 2, FRAIL scores were positively correlated with GDS‐15 scores (*r* = 0.19, *p* < 0.01) and EORTC sleep disturbances (*r* = 0.17, *p* < 0.01). GDS‐15 scores were correlated with EORTC sleep disturbance (*r* = 0.21, *p* < 0.01), EORTC pain severity (*r* = 0.33, *p* < 0.01), and EORTC pain interference (*r* = 0.37, *p* < 0.01). EORTC sleep disturbance was weakly correlated with EORTC pain severity (*r* = 0.21, *p* < 0.01) and EORTC pain interference (*r* = 0.17, *p* < 0.01). EORTC pain severity and pain interference were strongly correlated (*r* = 0.63, *p* < 0.01).

### 3.3 Analysis of the Mediating Effect of Sleep Disturbances Between Depressive Symptoms and Frailty

**FIGURE 2:**
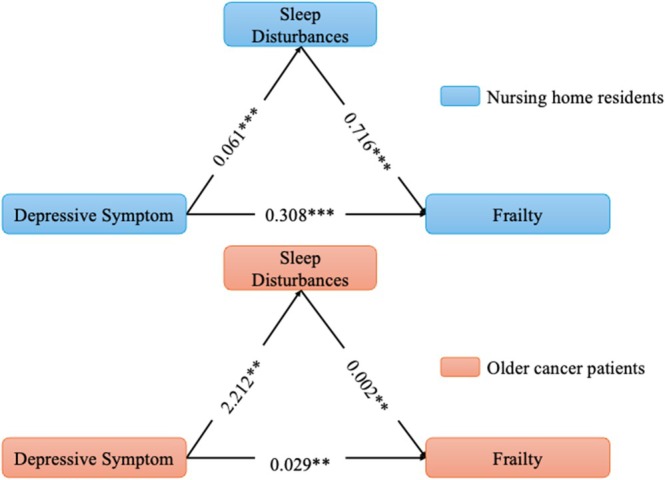
Mediation modeling results assessing the effect of depressive symptoms on frailty (*N* = 399). *p < 0.05, **p < 0.01, ***p < 0.001.

Figure 2 summarizes the regression paths from the mediation models. In both studies, depressive symptom scores were significantly associated with frailty scores. In Study 1, PHQ‐9 scores were positively associated with FRAIL‐NH scores (*β* = 0.308, *p* < 0.001). Additionally, PSQI scores showed a significant indirect association between PHQ‐9 scores and FRAIL‐NH scores, with an estimated effect size of 0.044. In Study 2, GDS‐15 scores were positively associated with FRAIL scores (*β* = 0.029, *p* < 0.01). EORTC sleep disturbance showed a significant indirect association between GDS‐15 scores and FRAIL scores, with an estimated indirect effect of 0.005. Results from both studies were consistent with a statistically significant indirect association through sleep disturbances (Figure 2).

### 3.4 Analysis of the Moderating Effect of Pain on the Mediating Effect of Sleep Disturbances

**FIGURE 3:**
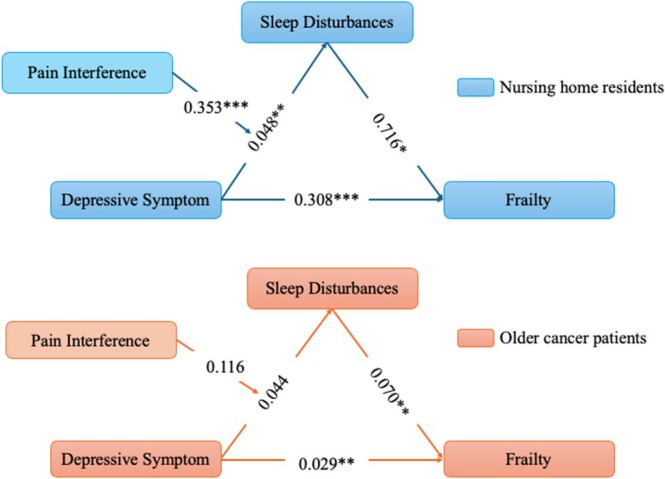
Moderated mediation modeling results assessing the effect of depressive symptoms on frailty. *p < 0.05, **p < 0.01, ***p < 0.001.

In Study 1, BPI pain interference rather than BPI pain severity moderated the association between PHQ‐9 scores and PSQI scores, indicating a moderated mediation effect. The PHQ‐9 × BPI pain interference interaction term was statistically significant (*β* = −0.011, SE = 0.005, *t* = −2.214, *p* < 0.05), indicating that the association between PHQ‐9 scores and PSQI scores varied by levels of pain interference. However, in Study 2, neither EORTC pain severity nor EORTC pain interference significantly moderated the association between GDS‐15 scores and sleep disturbance, and the moderated mediation effect was not supported (Figure 3).

## 4 Discussion

This study examined associations among study‐specific measures of depressive symptoms, sleep disturbances, pain, and frailty in older adults with and without cancer. Mediation analyses indicated a significant indirect effect of PHQ‐9 scores on FRAIL‐NH scores through PSQI scores in Study 1, and a significant indirect effect of GDS‐15 scores on FRAIL scores through EORTC sleep disturbance in Study 2. Moderated mediation analyses further showed that BPI pain interference moderated the association between PHQ‐9 scores and PSQI scores among older adults without cancer, whereas neither EORTC pain severity nor EORTC pain interference significantly moderated the corresponding association in older adults with cancer. Pain severity did not significantly moderate this pathway in either group.

The mediation analyses were consistent with Hypothesis 1, suggesting an indirect association through sleep disturbances in both nursing home residents and older patients with cancer. This finding aligns with previous research, which has consistently shown that depressive symptoms are closely associated with frailty and that sleep disturbances may contribute to both conditions (Bao et al. 2017; X. Liu, Wang, et al. 2021). The mutual relationship between sleep disorders and depressive symptoms, coupled with their shared underlying mechanisms (e.g., inflammation, neurochemical imbalances, and HPA axis dysregulation), is consistent with sleep disturbances as a potential pathway between depressive symptoms and frailty (Fang et al. 2019; Palmer and Alfano 2017). The high prevalence of sleep‐related issues among individuals with depressive symptoms (Tsuno et al. 2005) and the significant impact of sleep quality on cognitive and physical functioning in older adults further highlight the clinical relevance of assessing and managing sleep disturbances when addressing frailty risk in older adults (Jing et al. 2020; Liu et al. 2022).

The moderated mediation results partially supported Hypothesis 2 by indicating that pain interference moderated the association between depressive symptom scores and sleep disturbances in nursing home residents, but not in older patients with cancer. Nursing home residents may have higher burdens of chronic conditions and comorbidities, which could contribute to greater pain‐related interference and poorer sleep (Moore et al. 2012). In addition, variability in pain assessment and management resources in nursing homes may contribute to stronger coupling between pain interference and sleep disturbances (Lapane et al. 2020). In contrast, older patients with cancer may benefit from more structured pain management as part of oncology care, which could mitigate the moderating effect of pain interference on the relationship between depressive symptoms, sleep disturbances, and frailty (O'Regan et al. 2024). Furthermore, the nature of pain experienced by these two groups may differ, with nursing home residents more likely to experience generalized chronic pain and patients with cancer more likely to experience pain related to their disease and treatment (Myrenget et al. 2023; Tansug et al. 2021). This difference in pain types could influence how pain interference affects sleep disturbances and frailty.

### 4.1 Implications

The findings of this study have important implications for healthcare providers and policymakers working with older adults. The results suggest potential value in integrated care approaches that jointly consider depressive symptoms, sleep disturbances, and pain‐related interference in frailty management. Enhanced screening and assessment tools could be incorporated into routine care to identify at‐risk individuals and guide targeted interventions. Multidisciplinary teams, including mental health professionals, pain specialists, and sleep clinicians, may help design and implement coordinated interventions. For nursing home residents, prioritizing pain‐related interference and sleep problems may be particularly relevant, given the observed conditional associations in this group. For older patients with cancer, interventions targeting depressive symptoms and sleep disturbances may be more relevant, while pain interference did not show a significant moderating pattern in this sample.

### 4.2 Limitations and Future Research

We acknowledge that the study had several limitations: (1) The cross‐sectional design prevents causal inference regarding the temporal ordering among depressive symptoms, sleep disturbances, pain, and frailty. Longitudinal studies are needed to examine directionality. (2) This study relied on self‐reported data, which may affect the outcomes. Future research could incorporate objective or clinician‐rated assessments where feasible, such as actigraphy or sleep diaries for sleep, performance‐based measures for physical frailty, and clinical records for pain management. (3) These studies did not account for potential confounding factors such as medication use, comorbidities, and the severity of cancer, which could influence the relationships between depressive symptoms, sleep disturbances, pain, and frailty. Future research should aim to control for these variables to provide a clearer understanding of the underlying mechanisms. (4) Different instruments were used to assess key constructs across the two studies, which constrains measurement comparability and precludes formal measurement invariance testing. Although analyses were conducted in a study‐stratified manner and coefficients were standardized within each dataset to facilitate interpretation, these steps do not establish measurement equivalence. Future research could employ harmonized measurement tools across populations and settings. (5) Despite efforts to minimize confounding, residual bias from unmeasured contextual factors cannot be entirely excluded. Differences in health‐care systems, institutional practices, and cultural response patterns between Changsha and Hong Kong may have affected the observed associations. (6) While available health‐related characteristics were included, other clinically relevant variables such as comorbidity burden, medication use, and disease‐related indicators were not systematically collected. The absence of these measures may have influenced depressive symptoms, sleep disturbances, pain, and frailty, and should be considered when interpreting the findings. (7) While internal consistency reliability was assessed using Cronbach's alpha, the examination of other psychometric properties was constrained by the study design and the available dataset.

## 5 Conclusions

This study describes symptom‐frailty associations in older adults with and without cancer, showing both shared patterns and group‐specific differences. In both studies, results were consistent with a statistically significant indirect association between depressive symptoms and frailty through sleep disturbances. Among older adults without cancer, pain interference moderated the association between depressive symptoms and sleep disturbances, suggesting that the indirect association through sleep may vary by pain‐related interference. In older patients with cancer, neither pain severity nor pain interference significantly moderated the association between depressive symptom scores and sleep disturbances. These findings suggest that symptom‐focused approaches targeting depressive symptoms and sleep disturbances may be promising for frailty management, with additional attention to pain‐related interference in older adults without cancer.

## 6 Relevance for Clinical Practice

This study highlights the clinical importance of identifying and managing symptom clusters, particularly depressive symptoms, sleep disturbances, and pain, in older adults to mitigate frailty. The findings suggest that sleep disturbances serve as a key mediator between depressive symptoms and frailty, while pain interference plays a moderating role in non‐cancer populations. These insights highlight the need for integrated, symptom‐focused interventions tailored to the specific health profiles of older adults, especially those without cancer, to improve functional outcomes and quality of life.

## Author Contributions

**Tianxue Hou:** conceptualization, methodology, software, data curation, formal analysis, investigation, writing – original draft, writing – review and editing, visualization. **Denise Shuk Ting Cheung:** conceptualization, methodology, writing – review and editing. **Mu‐Hsing Ho:** conceptualization, writing – review and editing, validation, supervision. **Tongyao Wang:** writing – review and editing. **Chia‐Chin Lin:** conceptualization, writing – review and editing, supervision, validation, methodology.

## Funding

The authors have nothing to report.

## Ethics Statement

Ethical approvals for the study were attained from Xiangya School of Nursing, Central South University, and the Institutional Review Board of The University of Hong Kong/Hospital Authority Hong Kong West Cluster.

## Consent

The authors have nothing to report.

## Conflicts of Interest

The authors declare no conflicts of interest.

## Data Availability

The data that support the findings of this study are available from the corresponding author upon reasonable request.
